# Use of AgomiR and AntagomiR technologies to alter satellite cell proliferation *in vitro*, miRNA expression, and muscle fiber hypertrophy in intrauterine growth-restricted lambs

**DOI:** 10.3389/fmolb.2023.1286890

**Published:** 2023-11-03

**Authors:** M. A. Greene, G. A. Worley, A. N. S. Udoka, R. R. Powell, T. Bruce, J. L. Klotz, W. C. Bridges, S. K. Duckett

**Affiliations:** ^1^ Department of Animal and Veterinary Sciences, Clemson University, Clemson, SC, United States; ^2^ Clemson Light Imaging Facility, Clemson University, Clemson, SC, United States; ^3^ Department of Bioengineering, Clemson University, Clemson, SC, United States; ^4^ U. S. Department of Agriculture-Agricultural Research Service, Forage-Animal Production Research Unit, Lexington, KY, United States; ^5^ School of Mathematical and Statistical Sciences, Clemson University, Clemson, SC, United States

**Keywords:** miRNA, antagomiR, agomiR, proteomics, muscle fiber, hypertrophy, myoblasts

## Abstract

**Introduction:** microRNAs (miRNAs) are small non-coding RNAs that work at the posttranscriptional level to repress gene expression. Several miRNAs are preferentially expressed in skeletal muscle and participate in myogenesis. This research was conducted to alter endogenous miRNA expression in skeletal muscle to promote muscle hypertrophy.

**Methods:** Two experiments were conducted using mimic/agomiR or antagomir technologies to alter miRNA expression and examine changes in myoblast proliferation in vitro (experiment 1) and muscle hypertrophy *in vivo* (experiment 2). *In vitro* experiments found that antagomiR-22-3p and mimic-127 increased myoblast proliferation compared to other miRNA treatments or controls. These miRNA treatments, antagomiR-22-3p (ANT22) and agomiR-127 (AGO127), were then used for intramuscular injections in longissimus muscle.

**Results and discussion:** The use of antagomiR or mimic/agomiR treatments down-regulated or up-regulated, respectively, miRNA expression for that miRNA of interest. Expression of predicted target KIF3B mRNA for miR-127 was up-regulated and ACVR2a mRNA was up-regulated for miR-22-3p. ANT22 injection also up-regulated the major regulator of protein synthesis (mTOR). Proteomic analyses identified 11 proteins for AGO127 and 9 proteins for ANT22 that were differentially expressed. Muscle fiber type and cross-sectional area were altered for ANT22 treatments to transition fibers to a more oxidative state. The use of agomiR and antagomir technologies allows us to alter miRNA expression *in vitro* and *in vivo* to enhance myoblast proliferation and alter muscle fiber hypertrophy in IUGR lambs during early postnatal growth.

## 1 Introduction

Intrauterine growth restriction (IUGR) of the fetus alters muscle development and reduces birth weight (Yates et al., 2016; Greene et al., 2019; Greene et al., 2020; Hicks and Yates, 2021). Intrauterine growth restriction is associated with placental dysfunction and can be induced by hyperthermia (Yates et al., 2016), maternal undernutrition (Reed et al., 2014; Hoffman et al., 2016a; Hoffman et al., 2016b), and mycotoxin exposure (Greene et al., 2019; 2020; Britt et al., 2020). Impacts on muscle development depend on the timing of IUGR in relation to muscle fiber development and extent of the restriction. Reed et al. (2014) under-fed ewes from day 31 to parturition and reported reduced cross-sectional areas of muscle fibers at 3 months of age in lambs born to these underfed dams. Yates et al. (2014) induced hyperthermia in ewes from 40 to 95 days of gestation and found that myoblasts from the semitendinosus muscle of these fetuses had slower proliferation rates *in vitro*. Greene et al. (2019) exposed ewes to ergot alkaloids from days 35 to 85 and/or days 85 to 133 of gestation and reported smaller leg muscle weights with changes in miRNA expression. Alterations in fetal muscle development and growth due to IUGR can limit postnatal growth and alter carcass composition (Greenwood et al., 2005; Symonds et al., 2010).

miRNAs are a class of non-coding RNAs that regulate 60% of protein expression by post-transcriptional regulation (Friedman et al., 2009). The mechanism of action for miRNAs is to bind with the 3’ untranslated region of the target mRNAs and either inhibit translation or tag the mRNA for degradation (Eulalio et al., 2008; Hu and Coller, 2012). Several miRNAs (miR-1, -133a, -133b, -201, -208b, -486, and -499) have been identified as muscle-specific and present in high abundance in skeletal muscle tissue (Horak et al., 2016). The use of mimics/agomiRs or inhibitors/antagomiRs to overexpress or knockdown the expression of endogenous miRNAs, respectively, in C2C12 myoblasts or myoblasts has shown that these miRNA treatments can alter the proliferation and differentiation of muscle cells *in vitro* (Anderson et al., 2006; Chen et al., 2006; Crist et al., 2012; Antoniou et al., 2014; Qadir et al., 2014). The potential of miRNA treatments to regulate mRNA expression is being used in cancer treatments and has the potential to alter muscle growth (Rupaimoole and Slack, 2017); however, the use of miRNA treatments has not been tested in livestock species. Previous research documented changes in muscle fiber hypertrophy during prenatal and postnatal growth in lambs and identified key miRNAs that are involved in this process (Greene et al., 2022a). The objectives of this study were to examine the use of mimic/agomiR and antagomiR technologies to alter miRNA expression on 1) myoblast proliferation *in vitro* and 2) longissimus muscle hypertrophy *in vivo* during early postnatal growth in IUGR lambs.

## 2 Materials and methods

The use of animals was approved by the Clemson University Institutional Animal and Care Committee (AUP 2019-0069 and AUP-2019-0078).

### 2.1 Experiment 1: *in vitro* screening of miRNAs

Suffolk ewes (n = 4) were mated to Texel ram (Texel muscled; GeneSeek) and confirmed pregnant with twins using transabdominal ultrasound (BCF Easi-Scan portable ultrasound, BCF Technologies, Rochester, MN, United States). Ewes went to term, and male lambs (n = 4) were terminated at 2 days of age. Immediately after euthanization, the longissimus thoracis et lumborum muscle was removed and placed in Hank’s balanced salt solution containing 5% penicillin/streptomycin. Muscle samples were transported to the cell culture laboratory for the isolation of primary satellite cells. Muscle was finely minced under a laminar flow hood and digested using pronase E (1.5 mg/mL) according to the work of Li et al. (2009). The mechanical trituration method described by Danoviz and Yablonka-Reuveni (2012) was utilized after digestion and pelleting to allow maximal satellite cell recovery. At the end of the isolation procedure, the pre-plating technique of Gharaibeh et al. (2008) was used to further purify the satellite cell population by allowing the fibroblasts and myoblasts to plate down within 24 h and transferring the supernatant containing the satellite cells to another flask. The satellite cells were assessed for purity using immunofluorescence PAX7 staining, and the population was 98.5% pure (Greene et al., 2022a; Greene et al., 2022b; Supplementary Figure S1). The purity of the cell population was calculated as the total number of cells expressing PAX7 divided by the total number of nuclei counted. PAX7 is the marker for satellite cells and is expressed throughout quiescence and following activation during proliferation (Buckingham and Relaix, 2007). The use of PAX7 to identify the purity of satellite cell cultures has been previously established (Oustanina et al., 2004).

Satellite cells were activated by passaging using trypsin every 4 days for two passages to produce myoblasts for *in vitro* experiments (Motohashi et al., 2014). Myoblasts were cultured to assess the loss of function for four miRNA candidates (miR-29a, -22-3p, −133, or −27a) using antagomiR (Creative Biogene, Shirley, NY; Table 1) technologies or gain of function for two miRNAs (miR-127 and -299-5p) using mimic (Invitrogen, Thermo Fisher Scientific, Waltham, MA; Table 1) technologies during proliferation. Initial examination of miRNA inhibitors (Thermo Fisher) was found unsatisfactory because they did not downregulate miRNA expression *in vitro*, and therefore, antagomiR technologies were used for all loss-of-function assays. Cells were plated in 0.1% gelatin-coated 24-well plates at 20,000 cells/well (Danoviz and Yablonka-Reuveni, 2012). Cultures were allowed to reach ∼60% confluence prior to transfection and growth media [Dulbecco’s modified Eagle’s medium high glucose 4.5 g/L (DMEM; Gibco, Thermo Fisher), 20% fetal bovine serum (FBS; Avantor, VWR, Radnor, PA), 1% penicillin/streptomycin (Corning, VWR), and 0.1% gentamicin (VWR)] and were changed every 2 days. Following transfection, cultures were grown for 4 days in growth media.

**TABLE 1:** Sequences for miRNA, miRNA mimics, agomirs or antagomiRs, and TaqMan assays used in this experiment.

miRNA^a^	Sequence	Source
*oar*-miR-22-3p	AAG​CUG​CCA​GUU​GAA​GAA​CUG	miRBase, MI0025268
*bta-miR-22-3p*	AAG​CUG​CCA​GUU​GAA​GAA​CUG	miRBase, MI0005041
*TaqMan*-*bta*-miR-22-3p	AAG​CUG​CCA​GUU​GAA​GAA​CUG	Thermo Fisher, 4440886, 242214_mat
*AntagomiR-*22-3p	AAG​CUG​CCA​GUU​GAA​GAA​CUG	Creative Biogene, custom order
*oar*-miR-29a	UAG​CAC​CAU​CUG​AAA​UCG​GUU	miRBase, MI0014117
*hsa-miR-29a-3p*	UAG​CAC​CAU​CUG​AAA​UCG​GUU	miRBase, MI0020478
*TaqMan*-*hsa*-miR-29a	UAG​CAC​CAU​CUG​AAA​UCG​GUU	Thermo Fisher, 4,427,975, 000412
*AntagomiR-*29a	UAG​CAC​CAU​CUG​AAA​UCG​GUU	Creative Biogene, custom order
*oar*-miR-133	UUG​GUC​CCC​UUC​AAC​CAG​CUG​U	miRBase, MI0014122
*hsa*-miR-133a	UUG​GUC​CCC​UUC​AAC​CAG​CUG​U	miRBase, MI0000362
*TaqMan*-*hsa*-miR-133a	UUG​GUC​CCC​UUC​AAC​CAG​CUG​U	Thermo Fisher, 4,427,975, 000458
*AntagomiR-*133	UUG​GUC​CCC​UUC​AAC​CAG​CUG​U	Creative Biogene, custom order
*oar*-miR-27a	UUC​ACA​GUG​GCU​AAG​UUC​CGC	miRBase, MIMAT0030053
*hsa*-miR-27a-3p	UUC​ACA​GUG​GCU​AAG​UUC​CGC	miRBase, MI0000085
*TaqMan*-hsa-miR-27a	UUC​ACA​GUG​GCU​AAG​UUC​CGC	Thermo Fisher, 4,427,975, 000408
*AntagomiR-*27a	UUC​ACA​GUG​GCU​AAG​UUC​CGC	Creative Biogene, custom order
*oar*-miR-127	AUC​GGA​UCC​GUC​UGA​GCU​UGG​CU	miRBase, MIMAT0001415
*TaqMan*-oar-miR-127	AUC​GGA​UCC​GUC​UGA​GCU​UGG​CU	Thermo Fisher, 4440886, 008411_mat
*Mimic-oar-miR-127*	AUC​GGA​UCC​GUC​UGA​GCU​UGG​CU	Thermo Fisher, 4464066, MC10851
*AgomiR-oar-miR-127*	AUC​GGA​UCC​GUC​UGA​GCU​UGG​CU	Creative Biogene, custom order
*oar*-miR-299-5p	UGG​UUU​ACC​GUC​CCA​CAU​ACA​U	miRBase, MIMAT0019251
*hsa*-miR-299-5p	UGG​UUU​ACC​GUC​CCA​CAU​ACA​U	miRBase, MIMAT0002890
*TaqMan*-*hsa*-miR-299-5p	UGG​UUU​ACC​GUC​CCA​CAU​ACA​U	Thermo Fisher, 4,427,975, 000600
*Mimic-oar-miR-299-5p*	UGG​UUU​ACC​GUC​CCA​CAU​ACA​U	Thermo Fisher, 4464066, MC10330

^a^ Ovis aries (oar), *Bos taurus* (bta), or *Homo sapiens* (hsa) sequences from miRBase, the microRNA database, available at: https://www.mirbase.org/index.shtml.

*Ovis aries* miRNA sequences for miRNA candidates identified from previous sequencing results were acquired through miRBase (Table 1) and used for the initial screening for gain- or loss-of-function assays. Sequences were submitted to Creative Biogene for the custom microDOWN™ miRNA antagomiR synthesis of antagomiR-29a, -22-3p, −133, and −27a (500 nM). For miRNA mimics, sequences that matched *O. aries* sequences were identified and ordered (Thermo Fisher; Table 1). Mimics were transfected at 50 and 100 nM concentrations due to varying reports in the literature of the two different concentrations being used. RNAiMAX lipofectamine (Thermo Fisher) was used to transfect mimic or antagomiR treatments into myoblast cultures according to the manufacturer’s recommendations using Opti-MEM media (Thermo Fisher). RNAiMAX (negative control; NC) and Opti-MEM media (control; CON) were used as controls for antagomiR and mimic experiments. Myoblast proliferation was determined using Hoechst 33342 fluorochrome (Thermo Fisher) according to the manufacturer’s directions. Mimic and antagomiR experiments were run independently, and experiments were replicated using three replicate wells per sample by time. The intra-assay variance was <9.67%, and the inter-assay variance was <14.76%. On days 1 and 4 of the experiment, cells were collected for RNA extraction to examine miRNAs and predicted target mRNA expression as described in the following section.

### 2.2 Experiment 2: *in vivo* examination of antagomiR and agomiR treatments

*Experimental design:* Suffolk ewes (n = 24) were synchronized and mated to Texel rams (Texel muscled; GeneSeek). Ewes (n = 18) carrying twins, as confirmed by transabdominal ultrasound at gestational days (gds) 45–60, were used in this study. Ewes were fed 60% of NRC requirements (NRC, 2007) for total digestible nutrients and crude protein from day 86 to parturition to induce IUGR (Hoffman et al., 2016a; Hoffman et al., 2016b). The diet consisted of whole corn, fescue (endophyte-free) hay, and limestone (Table 2). Ewes were fed twice daily at 0600 and 1400. Ewe weights and blood samples were collected weekly prior to feeding β-hydroxybutyrate concentration using a handheld meter (FreeStyle Optium Neo Blood Glucose and Ketones Monitoring system, Abbott Laboratories). If ewes reached a severe ketone concentration (≥1.6 mmol/L; Araújo et al., 2020), ewes were given 0.23 kg of corn grain added to their basal ration until β-hydroxybutyrate concentrations dropped below the severe level. Ewes went to term, and lamb weights were collected at birth. One ewe aborted on gd98 and was removed from the study. Following birth, nutrient restriction ceased, and ewes were fed the same diet at 100% of NRC for lactating ewes. On day 2 of age, ewe lambs (n = 8/treatment) were randomly selected for testing of either agomiR-127 (AGO127; n = 8) or antagomiR-22-3p (ANT22; n = 8) by direct intramuscular injection into the longissimus muscle. AgomiR-127 (100 nM) or antagomiR-22-3p (500 nM) were custom synthesized by Creative Biogene (NY) for *O. aries* sequences obtained from miRBase. AgomiR and antagomiR compounds were reconstituted in phosphate-buffered saline (PBS) to achieve desired concentrations for a 0.5-mL injection volume. AgomiR and antagomiR technologies are chemically modified miRNA mimics or inhibitors that do not require transfection for *in vivo* use.

**TABLE 2:** Diet composition and nutrient intake during late gestation, gd86 to parturition.

Ingredient	Percent, DM basis
Corn, %	28
Fescue (E-) hay, %	71
Limestone, %	1
Dry matter intake, kg/d	1.29
Nutrient intake, kg/d	
TDN	0.82
Crude protein	0.135

**FIGURE 1:**
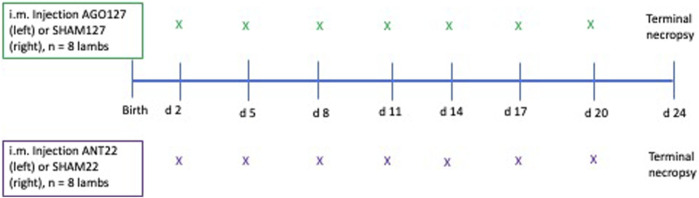
Diagram of injection sequence used in experiment 2. Lambs were randomly divided into two groups for agomiR-127 (AGO127, n = 8) or antagomiR-22-3p (ANT22, n = 8) intramuscular injections to alter endogenous miRNA expression. The first injection started on day 2 of age with the left longissimus muscle receiving the miRNA treatment (AGO127 or ANT22) and the right longissimus muscle receiving a SHAM (phosphate-buffered saline only) treatment. Lambs were terminated at 24 days of age and 4 days after the last i.m. injection for sample collection and measurements.

The left longissimus (LM) muscle was injected every 3 days, starting at the 10th rib and moving posteriorly by 1.27 cm at each injection to end at the 13th rib to avoid injecting in the same area repeatedly. A sham control treatment (SHAM127 or SHAM22) of PBS was administered following the same protocol for the right LM to serve as within-lamb control. A total of seven injections of agomiR or antagomiR were given per lamb, and the same number of injections were given to the sham (Figure 1). Lambs remained with their dams throughout the study. This injection strategy was based on *in vitro* results with mimic-127 and antagomiR-22-3p that found enhanced proliferation of myoblasts 4 days after miRNA treatment (experiment 1).

Lambs were harvested 4 days after the last injection, and LM tissue was collected from the injection site region (10th–13th rib) from the left (miRNA treatments) and right (SHAM) sides. Ribeye area was measured at the 10th rib, 13th rib, and at the hip. Samples of the injected region of both the right and left LM were individually snap frozen in liquid nitrogen and stored at −80°C for subsequent RNA, DNA, protein extraction, and proteomic analyses. Additional samples of LM were placed in a mold, covered using an optimal cutting temperature solution, snap frozen in liquid nitrogen, and stored at −80°C for subsequent histological examination.

### 2.3 RNA, DNA, and protein extraction

Total RNA was extracted from cell culture samples and muscle tissues from AGO127, ANT22, and SHAM injected regions using TRIzol reagent (Invitrogen, Thermo Fisher Scientific, Waltham, MA) according to the manufacturer’s instruction. The DNA-free kit (Ambion, Carlsbad, CA) was used according to the manufacturer to remove any genomic deoxyribonucleic acid (DNA) contamination from RNA samples. A NanoDrop1 spectrophotometer (Thermo Fisher) was used to quantify total RNA. RNA integrity numbers (RIN) were generated using an Agilent 4,200 TapeStation (Agilent Technologies, Santa Clara, CA), and all RIN values were above 9.5 for cells and above 7.0 for muscle tissues. Total RNA samples were stored at −80°C until further analysis.

Protein was extracted from LM samples using T-PER reagent (Thermo Fisher) as per the manufacturer’s recommendations. Protein amounts were quantified using the Pierce Coomassie Plus (Bradford) assay kit, and protein amounts were calculated on a per g of tissue basis. Measurements were made in triplicate. DNA was extracted from LM using the Extracta DNA Prep for PCR–Tissue kit (QuantaBio) as per the manufacturer’s recommendations. DNA was quantified using a NanoDrop1 spectrophotometer; measurements were made in triplicate, and the DNA amount was calculated on a per g of tissue basis.

### 2.4 miRNA RT-qPCR

miRNA sequences for *O. aries* were obtained through miRBase, and then, sequences were matched in the TaqMan assay database (Thermo Fisher; Table 1). Complementary DNA (cDNA) was synthesized using the TaqMan miRNA reverse transcription kit (Thermo Fisher; catalog no. 4366596). TaqMan small RNA assay kits (Thermo Fisher) were used for miR-29a (assay no. 000412; catalog no. 4427975), miR-22-3p (assay no. 242214_mat; catalog no. 4440886), miR-133 (assay no. 000458; catalog no. 4427975), miR-27a (assay no. 000408; catalog no. 4427975), miR-127 (assay no. 008411_mat; catalog no. 4440886), and miR-299-5p (assay no. 000600; catalog no. 4427975). snRNA U6 was selected as a housekeeping gene for the normalization of miRNA gene expression and the U6 snRNA TaqMan assay kit (assay no. 001973; catalog no. 4427975; Thermo Fisher). For miRNA expression analysis, miRBase was used to obtain miRNA sequences for *O. aries*. miRNA sequences were then matched to the TaqMan assay database (Thermo Fisher). The TaqMan miRNA reverse transcription kit (Thermo Fisher) was used to convert miRNA to cDNA. The TaqMan small RNA assay kits (Thermo Fisher) for miR-22-3p (assay no. 242214_mat; catalog no. 444886) and miR-127 (assay no. 008411_mat; catalog no. 4440886) were used to examine the expression of miRNA. U6 snRNA was selected as the housekeeping gene for the normalization of miRNA gene expression and the U6 snRNA TaqMan sssay kit (assay no. 001973; catalog no. 4427975; Thermo Fisher). The QuantStudio3 Real-Time PCR system and the TaqMan Fast Advanced Master Mix were used for qPCR according to the manufacturer’s instructions. The 2^−ΔΔCT^ method was used to normalize and calculate the fold change from control (Livak and Schmittgen, 2001), and results are expressed as the log2 fold change (log2FC).

### 2.5 mRNA RT-qPCR

Potential targets for the miRNAs that increased myoblast proliferation in culture (miR-22-3p, miR-127, and miR-299-5p) were identified for mRNA expression assays. Three software programs (miRDB, TargetScan, and TarBase) were used to identify potential targets for each miRNA. In addition, the literature was reviewed for published targets of these miRNAs in the muscle (Trendelenburg et al., 2009; Zhai et al., 2017; Wang et al., 2018; Yuan et al., 2018; Li et al., 2020; Yoshida and Delafontaine, 2020; Wang et al., 2022). Primer sets were made using the PrimerQuest™ Tool (IDT, Coralville, IA) for the predicted and published mRNA targets of each miRNA for RT-qPCR (Supplementary Table S1). For mRNA expression analysis, 1 ug of total RNA was converted to cDNA using QuantaBio qScript cDNA SuperMix (VWR) according to the manufacturer’s instruction and stored at −20°C. SYBR green (PerfeCTa SYBR Green SuperMix low ROX; QuantaBio) was used for performing qPCR according to the manufacturer’s instruction using a QuantStudio 3 Real-Time PCR System (Thermo Fisher). Several housekeeping genes (beta-actin [ACTB], glyceraldehyde-3-phosphate dehydrogenase [GAPDH], eukaryotic transcription initiation factor 3 subunit k [EIF3K], and ubiquitously expressed prefoldin-like chaperone [UXT]) were examined for normalization using RefFinder (Xie et al., 2012). Results showed that EIK3K and UXT were the most stable housekeeping genes, and the geometric mean of EIK3K and UXT was used for data normalization. The 2^−ΔΔCT^ method was used to normalize and calculate the fold change from control, and results are expressed as log-2 fold change (log2FC).

### 2.6 Proteomics

Crushed longissimus tissue was submitted to the IDEA National Resource for Quantitative Proteomics (http://idearesourceproteomics.org/) for processing, quantification, and analysis. Trypsin was used to digest the tissue samples, and tandem mass tag Carbamidomethyl C 57.021 was added to tryptic peptides. Samples were run by using orbitrap LC–MS, and DIA-MS samples were analyzed using Scaffold DIA (3.2.1). DIA-MS data files were converted to the mzML format using ProteoWizard (3.0.19254; Chambers et al., 2012). Analytic samples were aligned based on retention times and individually searched against an intermediate chromatogram library with a peptide mass tolerance of 25.0 ppm and a fragment mass tolerance of 25.0 ppm. Fixed modification considered was Carbamidomethyl C. Only peptides with charges in the range [2.3] and length in the range [6.30] were considered. Peptides identified in each sample were filtered by a percolator (3.01.nightly-13-655e4c7-dirty) to achieve a maximum FDR of 0.01. Individual search results were combined, and peptide identifications were assigned posterior error probabilities and filtered to an FDR threshold of 0.01 by a percolator (3.01.nightly-13-655e4c7-dirty; Käll et al., 2007; Käll et al., 2008a; Käll et al., 2008b). Peptide quantification was performed by Encyclopedia (1.12.31). For each peptide, the eight highest-quality fragment ions were selected for quantitation. Proteins that contained similar peptides and could not be differentiated based on MS/MS analysis were grouped to satisfy the principles of parsimony. Protein groups with a minimum of two identified peptides were thresholded to achieve a protein FDR less than 1.0%.

### 2.7 Muscle fiber histology

Longissimus muscle samples at the 12/13th ribs were collected at harvest, cryopreserved, and cryosectioned as described by Greene et al. (2022a). Two tissue sections per animal were used for type I/IIa/IIx myofiber typing. Cryosections of muscle samples were stained to identify type I/IIa/IIx myofibers using primary antibodies: MHC-slow type 1 mouse IgG2b (Developmental Studies Hybridoma Bank [DSHB] Cat# BA-F8, RRID:AB_10572253); MHC-type IIa mouse IgG1 (DSHB Cat# SC-71, RRID:AB_2147165); and MHC-type IIx mouse IgM (DSHB Cat# 6H1, RRID:AB_1157897) and secondary antibodies: Alexa Fluor 647 goat anti-mouse IgG2b (Thermo Fisher Scientific Cat# A-21242, RRID:AB_2535811); Alexa Fluor 546 goat anti-mouse IgG1 (Thermo Fisher Scientific Cat# A-21123, RRID:AB_2535765); and Alexa Fluor 488 goat anti-mouse IgM (Thermo Fisher Scientific Cat# A-21042, RRID:AB_2535711). Stained muscle sections were mounted in ProLong Gold (Cat #P36939, Invitrogen), and samples were imaged using a Leica DMi8 widefield microscope system (Leica Microsystems, Buffalo Grove, IL). To image proteins stained with Alexa Fluor 488 (type IIx fibers, depicted in green), we used a GFP filter cube (Ex/Em 455-495/505–555 nm); to image proteins stained with Alexa Fluor 546 (type IIa fibers, depicted in red), we used a Cherry filter cube (Ex/Em 540–580/592–668 nm); and to image proteins stained with Alexa Fluor 647 (type 1 fibers, depicted in magenta), we used a Y5 filter cube (Ex/Em 600-660/662–738 nm). Images were collected, exported, analyzed, and subjected to statistical analyses as outlined by Greene et al. (2022a).

### 2.8 Statistical analysis

In experiment 1, analysis of variance was performed to analyze myoblast proliferation and gene expression by miRNA treatment at each time point. In experiment 2, ewe body weights were analyzed using a mixed procedure of SAS (SAS Institute, 9.4) with gestation week in the model. Lamb body weights were analyzed using a mixed procedure of SAS with miRNA treatment, time, and two-way interaction in the model. A paired *t*-test was used to compare the treated (AGO127 or ANT22) longissimus to the control (SHAM127 or SHAM22) longissimus as an animal control for ribeye area, protein content, DNA content, miRNA and mRNA gene expression, and proteomics data. For histology, muscle fiber types and cross-sectional areas were analyzed using a mixed procedure of SAS to compare miRNA and SHAM in each animal by miRNA treatment. The histology model included sections and images as random effects in the model. Statistical significance was determined at *p* < 0.05 and trends at *p* < 0.10.

## 3 Results

### 3.1 Experiment 1

**FIGURE 2:**
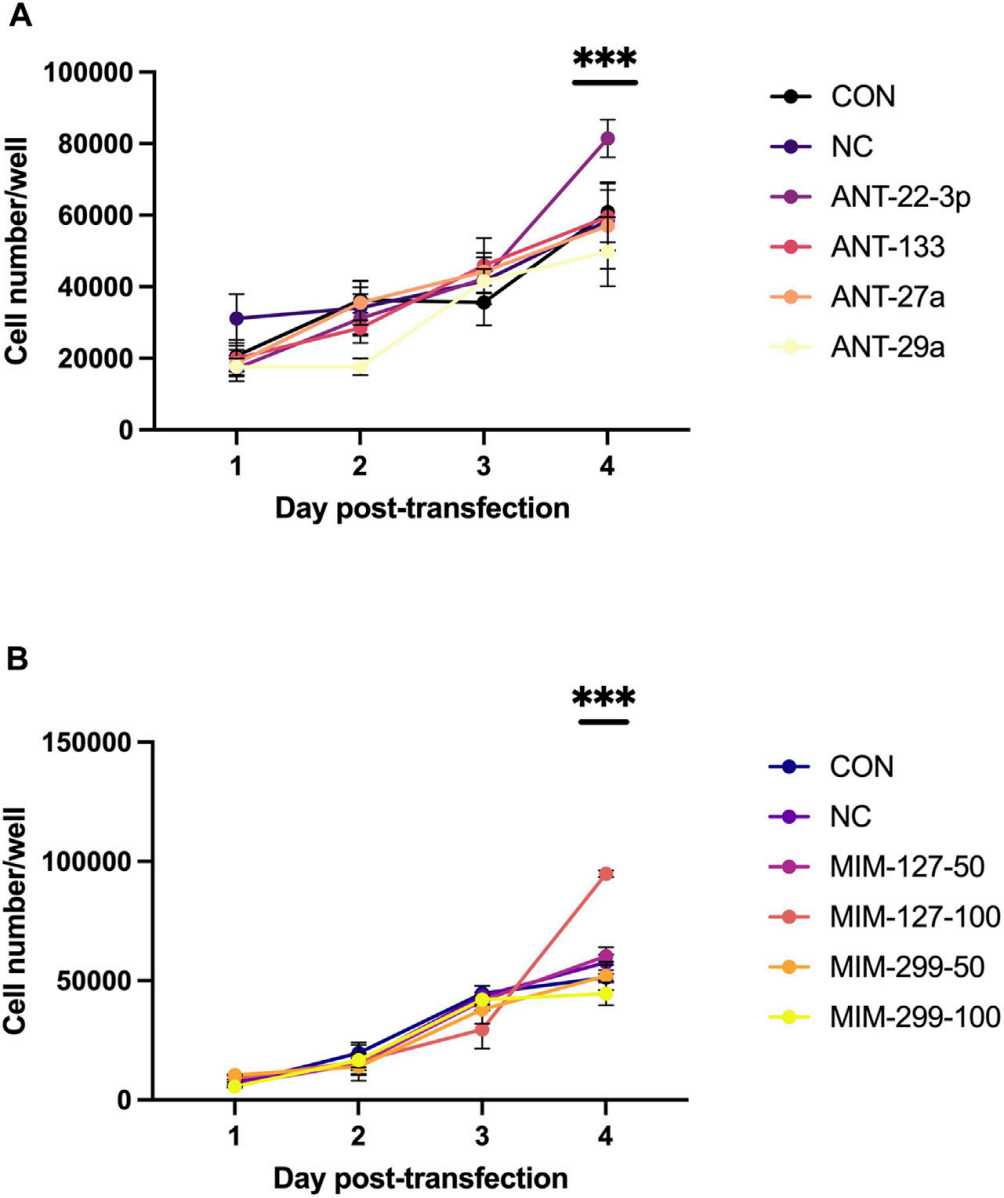
Myoblast proliferation over time in antagomiR (ANT-22-3p, −27a, −29a, and 133; 500 nM)-treated cultures **(A)** or mimic (MIM-127 and -299 at 50 or 100 nM)-treated cultures **(B)** compared to control or negative control in experiment 1.

**FIGURE 3:**
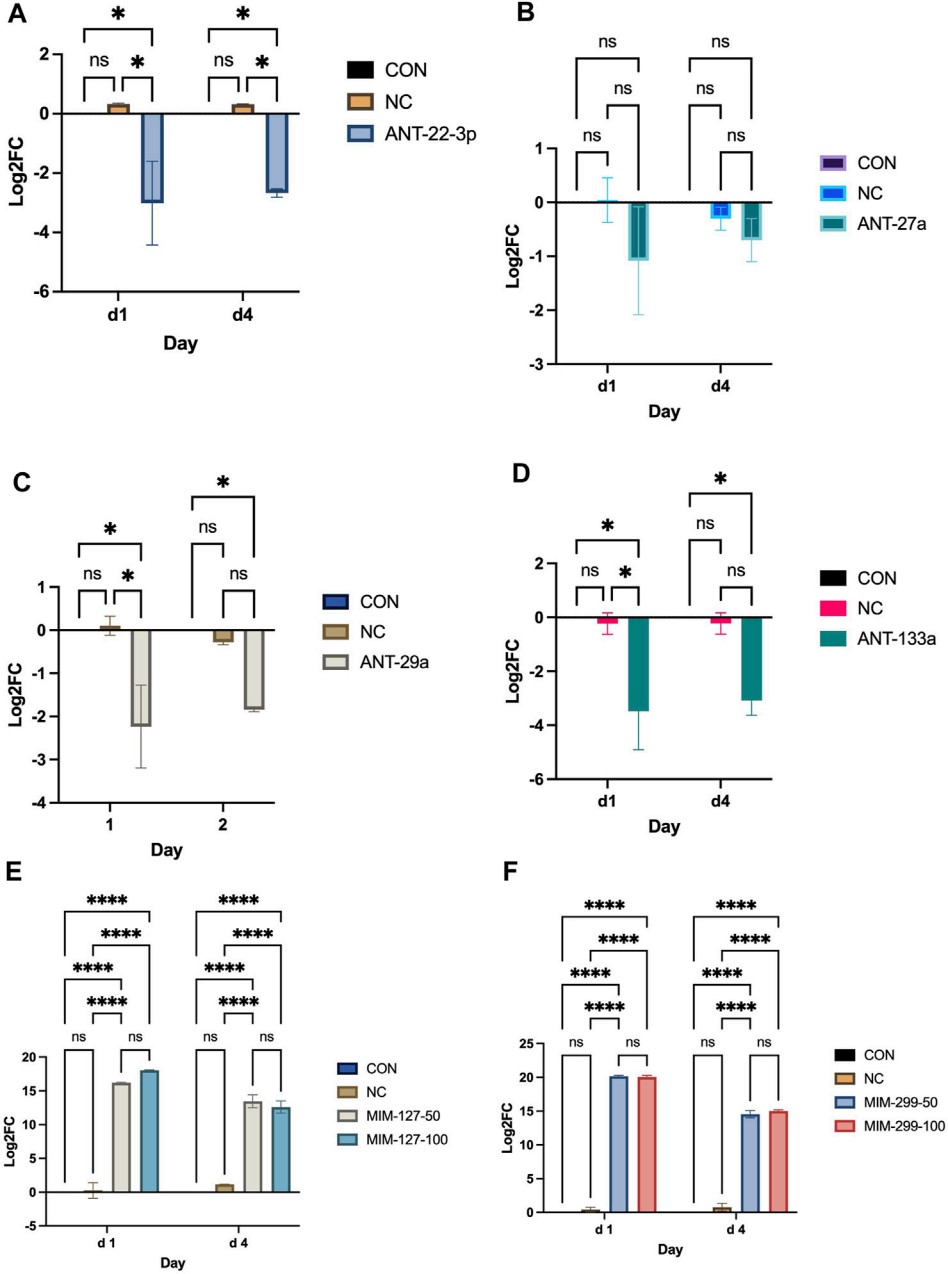
miRNA expression on days 1 and 4 post-transfection with antagomiRs (-22-3p, −27a, −29a, and −133; **(A–D)** and mimics (-127 or -299 at 50 and 100 mM; **(E, F)** in experiment 1.

Experiment 1 was conducted to examine miRNAs identified by sequencing as differentially expressed in the ovine longissimus muscle during early postnatal growth (Greene et al., 2022b) *in vitro* to identify miRNAs that enhance myoblast proliferation. Myoblast proliferation increased during the 4-day *in vitro* assay (Figure 2). On day 4, antagomiR-22-3p treatment had higher (*p* < 0.01) cell numbers compared to all other antagomiR treatments, control or negative control (Figure 2). AntagomiR-29a, −27a, and −133 treatments did not alter myoblast proliferation compared to the control or negative control. On day 4, mimic-127 at 100 nM concentration (MIM-127-100) had a greater (*p* < 0.01) myoblast cell number than other mimic treatments, control or negative control (Figure 2). Mimic-127 at 50 nM and mimic-299-5p at both concentrations did not alter myoblast proliferation in this experiment. The miRNA expression of antagomiR-treated cells was downregulated (*p* < 0.05) for ANT-29a, -22-3p, and −133 on days 1 and 4 of culture, indicating the efficacy of antagomiRs to inhibit the miRNA expression of that specific miRNA (Figure 3). The addition of mimics to the myoblast cultures upregulated (*p* < 0.0001) miRNA expression for each mimic treatment at both concentrations (50 and 100 nM), indicating the efficacy of mimics to increase miRNA expression (Figure 3).

**FIGURE 4:**
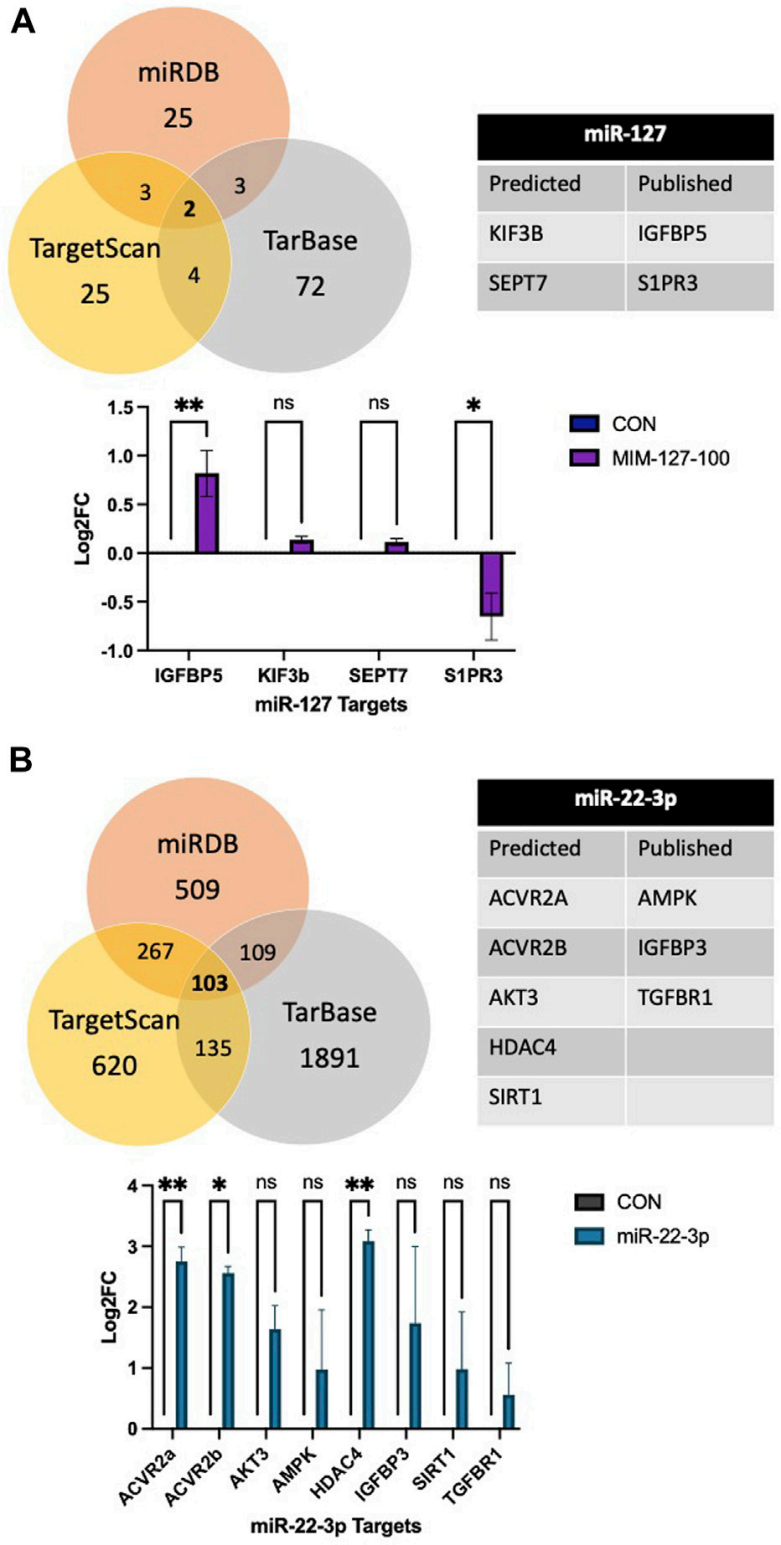
Predicted mRNA targets of miR-127 **(A)** and miR-22-3p **(B)** from software programs, published targets, and mRNA expression of targets.

There is no software prediction program for the *O. aries* species to help identify targets of each miRNA. Prediction of targets for miR-22-3p and −127 was examined using three software prediction programs (miRDB, TargetScan, and TarBase) using human sequences. For miR-127, the three software programs all predicted two genes, KIF3B and SEPT7, as predicted targets and IGFBP5 and S1PR3 were reported targets (Figure 4). Expressions of mRNA targets (KIF3B, SEPT7, IGFBP5, and S1PR3) did not differ between mimic-127-100 and controls. For miR-22-3p, there were 103 predicted targets from the three software programs that aligned (Figure 4B). From this list of predicted targets, five genes that had known roles in myogenesis or cell proliferation were chosen for mRNA expression (ACVR2a, ACVR2b, AKT3, HDAC4, and SIRT1). We also examined the published literature on miR-22-3p and found potential targets that had been discovered (AMPK, HDAC4, IGFBP3, SIRT1, and TGFBR1). These predicted and published targets were examined by qPCR in day 4 myoblast culture samples from antagomiR-22-3p treatments. Expressions of HDAC4, ACVR2a, and ACVR2b were upregulated (*p* < 0.05) compared to control. Expressions of other predicted targets (AKT3, AMPK, IGFBP3, SIRT1, and TGFBR1) did not differ between antagomiR-22-3p-treated cells and controls.

### 3.2 Experiment 2

Experiment 2 was conducted to examine miRNAs identified in experiment 1 as enhancing myoblast proliferation for use *in vivo* to examine changes in skeletal muscle hypertrophy in IUGR lambs. During nutrient restriction, ewe body weight (BW) did not change (*p* > 0.05) from the start (gd86) of restriction to parturition (Table 3). The average length of gestation was 145.3 days. Total lamb birth weight averaged 9.18 kg per ewe or 11.41% of ewe BW. The ketosis level was monitored throughout the study by monitoring β-hydroxybutyrate concentrations. Severe ketosis levels (≥1.6 mmol/L β-hydroxybutyrate) were observed starting at gd127 through parturition for 47% of the ewes. The greatest incidence (35%) of ewes in severe ketosis was between gd127 to gd133. Lamb body weight increased (*p* < 0.0001) during the treatment period for both AGO127/SHAM127 and ANT22/SHAM22 (Table 4). There were no differences (*p* > 0.20) in the body weight or average daily gain between the miRNA treatment groups.

**TABLE 3:** Changes in ewe body weight and incidence of severe ketosis during the nutrient restriction (gd86 to parturition) period of this study.

Gestation day	Ewe weight, kg	Severe ketosis^a^, no.
Ewe (n = 17)		
Week 1 (gd86–92)	90.2	0
Week 2 (gd93–99)	89.1	0
Week 3 (gd100–106)	87.8	0
Week 4 (gd107–113)	86.9	0
Week 5 (gd114–120)	88.1	0
Week 6 (gd121–127)	89.5	1
Week 7 (gd128–134)	90.4	6
Week 8 (gd135–141)	88.9	2
Week 9 (gd142–parturition)		1
SEM		
**Gestation length, d**	145.3 ± 2.02	
Total lamb weight, kg	9.18 ± 0.92	
Total lamb weight, % of ewe BW	11.41 ± 1.15	

^a^ Severe ketosis: β-hydroxybutyrate concentrations ≥1.6 mmol/mL.

**TABLE 4:** Body weight of lambs used in miRNA treatments, agomiR-127 (AGO127) or antagomiR-22-3p (ANT22). Lamb served as its own control with miRNA treatment on the left side and SHAM treatment on the right side of the longissimus muscle in each animal for a 24-day period.

Body weight, kg	AGO127/SHAM127	ANT22/SHAM22	SEM
n	8	8	
d 0 (birth)	4.89	4.60	0.21
d 2	5.13	5.12	0.22
d 5	6.35	6.13	0.26
d 8	7.34	7.23	0.33
d 11	8.56	8.53	0.35
d 14	9.46	9.52	0.43
d 17	10.27	10.63	0.51
d 20	10.79	11.31	0.60
d 24	11.62	12.49	0.71
Average daily gain, g/d	280.2	328.7	0.027

The ribeye area of the injected region did not differ (*p* > 0.05) for AGO127 or ANT22 compared to their respective SHAM (Table 5). However, the weight of the injected region was heavier (*p* < 0.05) for both AGO127 and ANT22 compared to their respective SHAM. The DNA, RNA, and protein content of the LM did not differ (*p* > 0.05) between AGO127 and SHAM127. For ANT22, DNA and protein content did not differ, but RNA content was increased (*p* < 0.01) for ANT22 compared to SHAM22. Ratios between RNA to DNA and RNA to protein were elevated (*p* < 0.01) for ANT22 than for SHAM22.

**TABLE 5:** Longissimus muscle (LM) characteristics for miRNA treatments, Agomir-127 (AGO127) or AntagomiR-22-3p (ANT22), and their respective SHAM within animal control.

Injected region of LM	SHAM127	AGO127	SEM	*p*-value
n	8	8		
Ribeye area, cm^2^	10.81	10.06	0.097	0.28
Weight, g	49.87	55.97	1.78	0.011
DNA, ug/g	7107.65	7592.13	351.8	0.22
RNA, ug/g	535.91	448.40	129.0	0.52
Protein, ug/g	49400.74	54780.41	3288.9	0.15
Protein:DNA	6.97	7.31	0.441	0.47
RNA:DNA	0.076	0.0597	0.0157	0.33
RNA:Protein	0.0114	0.00825	0.00271	0.29

Injected region of LM	SHAM22	ANT22	SEM	*p*-value
n	8	8		
Ribeye area, cm^2^	10.56	10.68	0.064	0.77
Weight, g	55.66	60.13	1.62	0.021
DNA, ug/g	7515.60	8229.94	503.5	0.199
RNA, ug/g	368.59	610.68	49.02	0.0017
Protein, ug/g	57488.73	55498.37	1778.8	0.300
Protein:DNA	7.78	6.78	0.433	0.054
RNA:DNA	0.0498	0.0753	0.0051	0.0016
RNA:Protein	0.0065	0.0111	0.00079	0.0006

**FIGURE 5:**
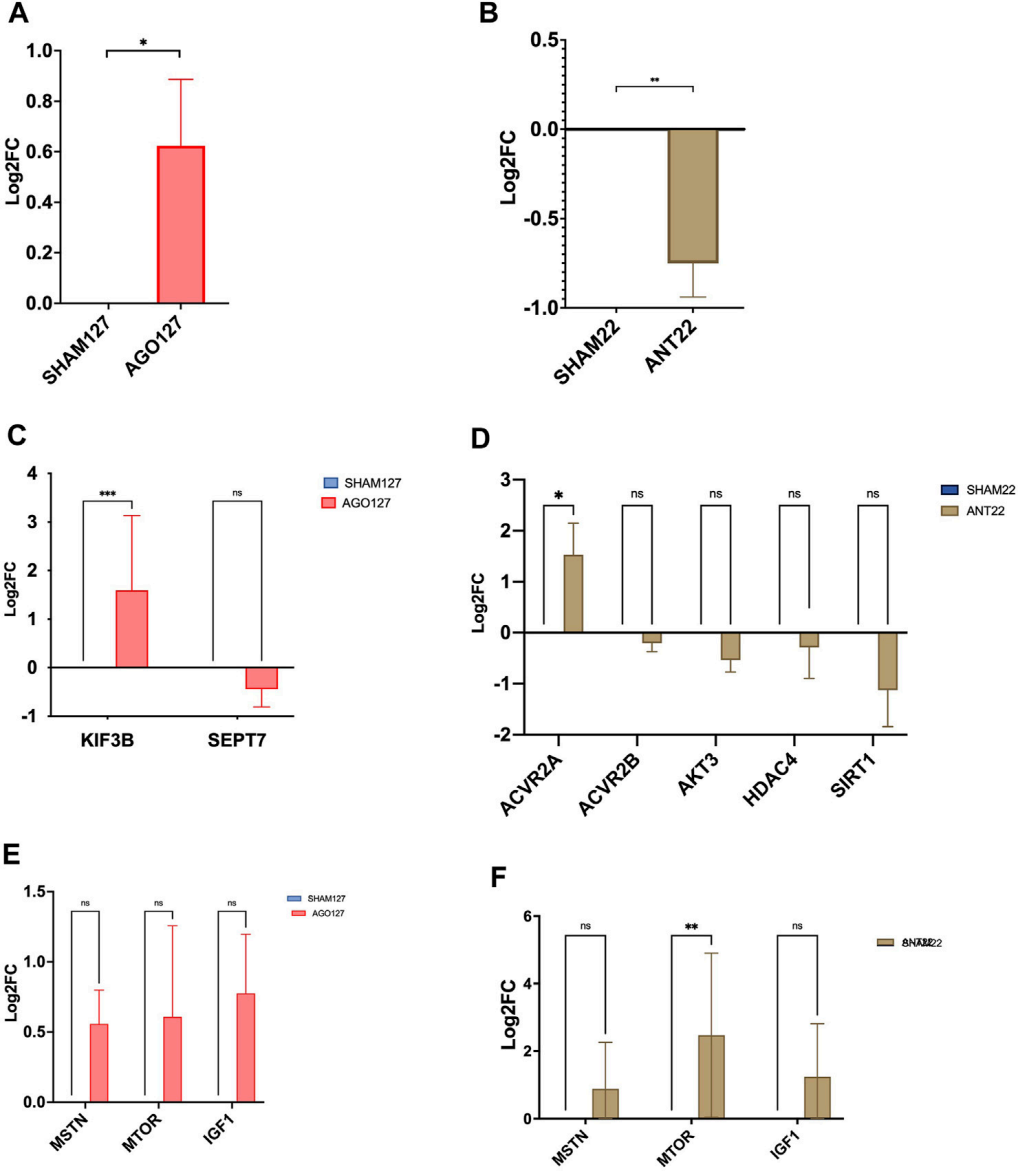
miRNA expression in AGO127 muscle **(A)** or ANT22 muscle **(B)** *versus* their respective SHAMs in experiment 2. mRNA expression of predicted targets for AGO127 **(C)** and ANT22 **(D)** treatments and major regulators of protein synthesis **(E, F)**.

Treatment of the longissimus with AGO127 increased (*p* < 0.01) miR-127 expression when compared to SHAM127 samples (Figure 5). ANT22 treatment decreased (*p* < 0.01) the expression of miR-22-3p compared to SHAM22 samples. Predicted target, KIF3B, was upregulated (*p* < 0.001) in AGO127 compared to SHAM127. Expressions of MTOR, MSTN, or IGF1 were not altered (*p* > 0.05) by AGO127 expression. ACVR2A expression was upregulated (*p* < 0.05) by ANT22 treatment. Expressions of other mRNA targets (SIRT1, ACVR2B, AKT3, and HDAC4) were not altered (*p* > 0.05) for ANT22-treated samples. Treatment with ANT22 upregulated (*p* > 0.05) MTOR expression but did not alter (*p* > 0.05) the expression of MSTN and IGF1.

AGO127 treatment increased (*p* < 0.05) the cross-sectional area of type I fibers when compared to SHAM127; however, the type I fiber number tended to be reduced for AGO127-treated samples (data not shown). Type IIa fibers did not differ (*p* > 0.05) in the area or number between AGO127 and SHAM127. Intermediate fibers, type IIax, had a larger (*p* < 0.01) cross-sectional area for AGO127 than SHAM127. The number of type IIax fibers did not differ (*p* > 0.05) by AGO127 treatment. The total number of fibers examined for AGO127 and SHAM127 samples did not (*p* < 0.05) differ by treatment. The expression of myosin heavy-chain isoforms, MYHC1, MYHC2A, and MYHC2X, was not altered (*p* > 0.05) by AGO127 treatment.

**FIGURE 6:**
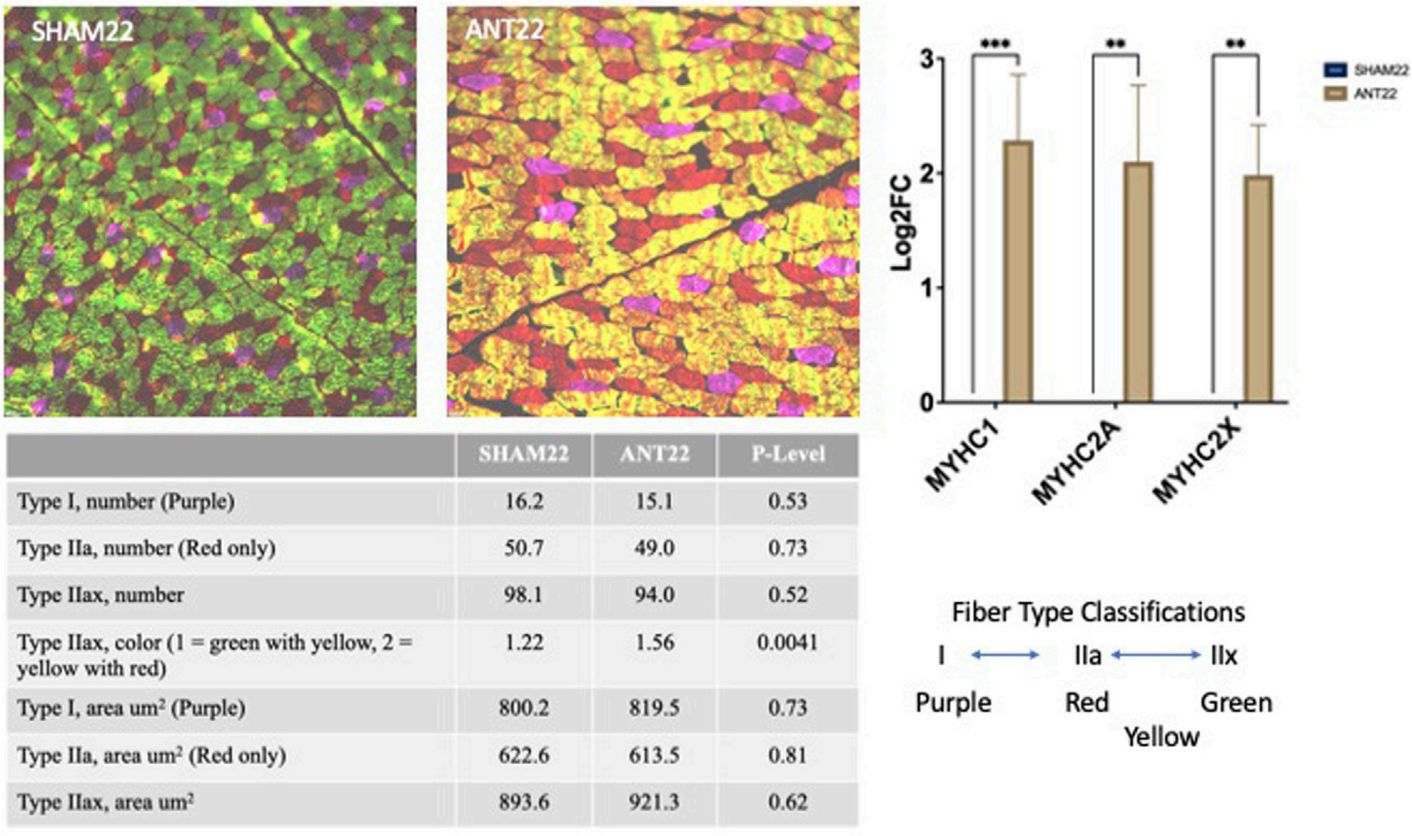
Muscle fiber type and cross-sectional area of the longissimus muscle with SHAM22 or ANT22 treatment were measured in experiment 2. Two tissue sections per animal were used to identify myosin heavy-chain isoforms (MyHC): MyHCI = purple, MyHCIIa = red, MyHCIIx = green, and fiber staining for both MyHCIIa and MyHCIIx = yellow. Images were collected and analyzed using ImageJ to measure cross-sectional area and count numbers, which were subjected to statistical analyses.

Type I fiber number and fiber cross-sectional area were not altered (*p* > 0.05) by ANT22 treatment (Figure 6). ANT22 treatment did not alter (*p* > 0.05) the type IIa fiber number or fiber cross-sectional area. During the examination of muscle fiber histology, there were noticeable differences in the coloring patterns of intermediate fibers (type IIax). Therefore, intermediate fibers were classified as type IIax with green with yellow patterns or type IIax with yellow with red patterns to denote differences in metabolic types that were observed. The ratio of type IIax muscle fibers based on the color (1 = green with yellow; 2 = yellow with red) was greater (*p* < 0.01) for ANT22 compared to SHAM22. This indicates that more muscle fibers were transitioning toward a more oxidative state for those staining with yellow/red color. The cross-sectional area of the type IIax fibers did not differ (*p* > 0.05) for type IIax or by color classification. The expression of myosin heavy-chain isoforms, MYHC1, MYHC2A, and MYHC2X, was upregulated (*p* < 0.001) in ANT22-treated samples compared to SHAM22.

**FIGURE 7:**
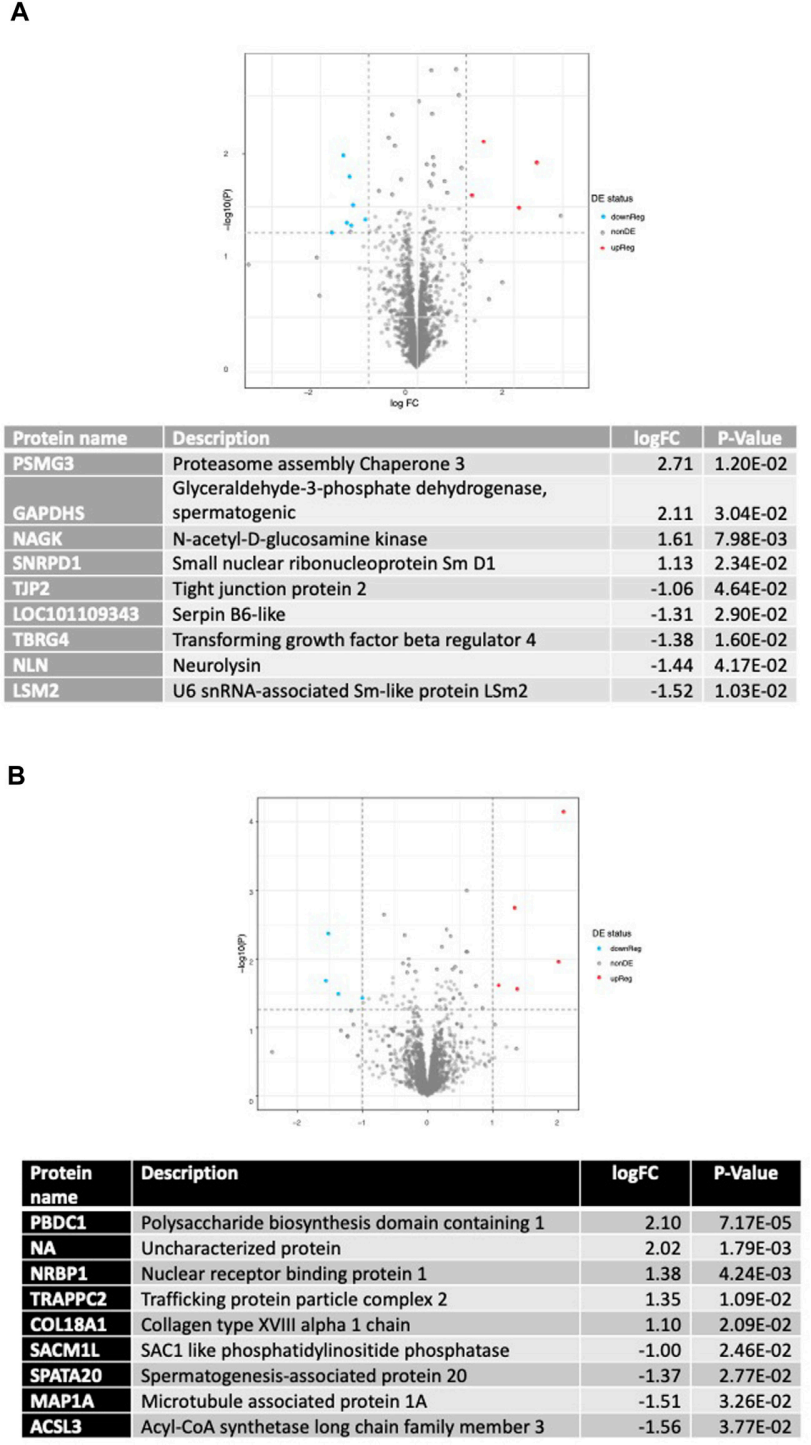
Differentially expressed proteins found using proteomic analysis of AGO127 vs. SHAM127 **(A)** or ANT22 vs. SHAM22; **(B)** longissimus muscle samples with *p* < 0.05 and log2FC > 1.

Proteomics analysis for AGO127 vs. SHAM127 is displayed in Figure 7. Eleven proteins were differentially expressed (*p* < 0.05) with a Log2FC > |1|, six proteins were found to be upregulated [proteasome assembly chaperone 3, glyceraldehyde-3-phosphate dehydrogenase (spermatogenic), N-acetyl-D-glucosamine kinase, small nuclear ribonucleoprotein Sm D1, RING-type E3 ubiquitin transferase, and small nuclear ribonucleoprotein Sm D1], and five proteins were downregulated (tight junction protein 2, Serpin B6-like, transforming growth factor beta regulator 4, neurolysin, and U6 snRNA-associated Sm-like protein LSm2). For ANT22, nine proteins were found to be differentially expressed (*p* < 0.05) with a Log2FC > |1| (Figure 7). Five proteins were upregulated (polysaccharide biosynthesis domain-containing 1, nuclear receptor-binding protein 1, trafficking protein particle complex 2, collagen type XVIII alpha 1 chain, and an uncharacterized protein), and four proteins were downregulated (SAC1-like phosphatidylinositide phosphatase, spermatogenesis-associated protein 20, microtubule-associated protein 1A, and acyl-CoA synthetase long-chain family member 3) with ANT22 treatment.

## 4 Discussion

miRNA sequencing of skeletal muscle during development showed that early postnatal growth represents a critical time-period for the involvement of miRNAs in skeletal muscle hypertrophy (Greene et al., 2022a). In this study, we evaluated several miRNAs that were identified as being upregulated (miR-22-3p, -27a, -29a, and -133; *p* < 1.0^e −10^) or downregulated (miR-127 and -299-5p; *p* < 1.0^e−10^) during early postnatal muscle hypertrophy from the sequencing results (Greene et al., 2022a). Mimics were chosen to upregulate the miRNA expression of miR-127 and miR-299 back to prenatal levels, whereas antagomiRs were used to downregulate the expressions of miR-22-3p, miR-27a, miR-29a, and miR-133 to lower levels observed during prenatal growth. Satellite cell populations are highly proliferative during late gestation and early postnatal life but decline with advanced maturity (Mesires and Doumit, 2002). Myoblast proliferation was greater for MIM-127-100 on day 4 compared to other mimic treatments, control or negative control. In the antagomiR experiment, myoblast proliferation was greater on day 4 compared to other antagomiR treatments, control or negative control. Others have shown that miRNA mimics or inhibitors can be used to alter myoblast proliferation in C2C12 mouse immortalized cell lines (Anderson et al., 2006) or satellite cells isolated from mice (Crist et al., 2012), pigs (Zhu et al., 2019), or sheep (Zhao et al., 2016; Zhang et al., 2018). The other antagomiRs (-29a, -27a, or -133) examined in this study did not alter myoblast proliferation. In contrast, others have shown miR-29a inhibition with antagomiR (Greene et al., 2022a; 300 nM) or an inhibitor (Wu et al., 2020, 200 nM) alters myoblast proliferation, whereas mir-27a overexpression (Huang et al., 2012; Chen et al., 2014) and miR-1/-133 overexpression (Zhang et al., 2012) alter differentiation when used at different concentrations or via different transfection compounds or technologies. In our study, miRNA expression was up-regulated with mimic treatments and downregulated with antagomir treatments *in vitro*, which show the efficacy of these compounds to alter endogenous miRNA expression. Based on these *in vitro* results, we proceed to experiment 2 to further examine the upregulation of miR-127 and downregulation of miR-22-3p on *in vivo* muscle hypertrophy during early postnatal growth.

In experiment 2, ewes were nutrient-restricted during late gestation which resulted in no weight gain, and 47% of ewes displayed severe ketosis. Over 80% of fetal growth occurs during late gestation in twin-bearing ewes (Rattray et al., 1974), and IUGR during this time can reduce muscle fiber cross-sectional areas and miRNA expression (Greene et al., 2019; Greene et al., 2022b). In order to alter miRNA expression *in vivo*, we used agomiRs and antagomiRs that were custom synthesized to *O. aries* miRNA sequences and do not require transfection *in vivo*. AgomiRs are miRNA mimics that have been chemically modified to enhance cellular uptake and stability. miRNA mimics are chemically modified double-stranded RNA molecules that mimic specific endogenous miRNAs and contribute to the downregulation of target mRNAs (Wang, 2011). miRNA inhibitors function by binding to specific mature endogenous miRNAs with a reverse complement and preventing miRNA-induced silencing complex cleavage and targeting of mRNAs (Esau, 2008). AntagomiRs are single-stranded RNAs with a cholesterol conjugate and are complementary to the target miRNA (Krützfeldt et al., 2005). The antagomiR binds with the target miRNA and prevents it from binding with mRNA and disrupting translation; once bound, the miRNA target is unavailable to the cell. Limited research is available in livestock species on the use of agomiR or antagomiR technologies to alter miRNA expression in the skeletal muscle. For this study, we designed the injections to follow the *in vitro* study in which myoblast proliferation was increased 4 days after miRNA treatment. *In vitro* results showed that the miRNA expression was altered at day 4 but the fold change in expression was lower than at day 1. We injected every 72 h during this 21-day study in an attempt to keep miRNA expression at elevated (AGO127) or decreased (ANT22) expression and stimulate myoblast proliferation *in vivo*. Intramuscular injections of AGO127 were effective in upregulating miR-127 expression in the longissimus muscle, whereas intramuscular injections of ANT-22-3p downregulated the expression of miR-22-3p. These results demonstrate that the use of intramuscular agomiR or antagomiR injections alters endogenous miRNA expression levels *in vivo*. The use of agomiR or antagomiR technologies or repeated injections did not alter the growth of these lambs. The ribeye area of the injected region was not altered during this short-term study; however, changes in muscle weight and RNA content were observed. Both AGO127 and ANT22 treatments increased the weight of the injected region compared to their respective SHAM. The use of ANT22 increased the RNA content and the ratios of RNA to DNA and RNA to protein.

miRNAs can have multiple mRNA targets, and these targets depend on the species, tissue, or cell line (Huang et al., 2010; Chipman and Pasquinelli, 2019). These variations underline the importance of studying individual miRNA treatments in species and cell types of interest. miR-127 is predicted to alter KIF3B and SEPT7 expression; however, we did not find any changes in the mRNA expression of KIF3B or SEPT7 in MIM-127-treated cells *in vitro*. The intramuscular injection of AGO127 increased the mRNA expression of the target KIF3B, which is associated with skeletal muscle contractile structures such as the sarcoplasmic reticulum and the transverse tubules (Gönczi et al., 2022). The expression of SEPT7 was not altered *in vivo* with the intramuscular injection of AGO127. Li et al. (2020) found that the overexpression of miR-127-3p in C2C12 cells inhibited proliferation and targeted SEPT7 for downregulation. Others reported that miR-127 overexpression enhanced the differentiation of C2C12 cells by regulating the expression of spingosine-1-phosphate receptor 3 (S1PR3), a target of mir-127 (Zhai et al., 2017). In pigs, miR-127 and -299 were both identified as being downregulated in adult pigs (Chen et al., 2020).

For miR-22-3p, the three software programs agreed on over 100 mRNAs that may be targets of miR-22-3p, including ACVR2a or 2b, AKT3, HDAC4, and SIRT1. ACVR2a and ACVR2b are type II activin receptors for many of the transforming growth factor beta (TGFB) superfamily, which includes myostatin (MSTN), a negative regulator of myogenesis. In experiment 1 (*in vitro*), ANT22 treatment upregulated ACVR2a, ACVR2b, and HDAC4 expression compared to controls. In experiment 2 (*in vivo*), ANT22 treatment upregulated AVCR2A. Mutations in the myostatin gene are responsible for the double-muscled condition in Piedmontese and Belgian Blue cattle (McPherron and Lee, 1997) and Texel sheep (Clop et al., 2006). Knockdown of ACVR2a, ACVR2b, or MSTN alone or in various combinations has been shown to alter muscle mass in chickens (Bhattacharya et al., 2019). These authors found that the knockdown of ACVR2A and 2B increases muscle mass; however, silencing ACVR2B had a larger effect on muscle growth in chickens. Silencing of AKT3 with siRNA inhibits C2C12 cell proliferation while also promoting differentiation (Wei et al., 2013). SIRT1 is involved in muscle repair from injury, and ablation of SIRT1 impairs muscle function (Myers et al., 2019). Transfection of miR-22hg, the precursor for miR-22-3p, promoted C2C12 differentiation by inhibiting its target, HDAC4, which upregulates MEF2C (Li et al., 2021). Additionally, HDAC4 works through transcriptional regulation, cell cycle progression, and developmental events. The overexpression of miR-22-3p in primary skeletal muscle cells from Hu sheep promoted differentiation by targeting IGFBP3 (Wang et al., 2022). In C2C12 myoblasts, the overexpression of miR-22 decreased proliferation and promoted differentiation through the TGFBR1/SMAD3 pathway (Wang et al., 2018).

Muscle hypertrophy occurs when muscle protein synthesis exceeds muscle protein degradation. Muscle protein synthesis is controlled by two major signaling pathways that work as negative (MSTN, ACVRI/II) or positive (IGF1/PI3K/AKT) regulators of mammalian targets of the rapamycin (mTOR) complex (Schiaffino et al., 2013). IGF1 binds to its receptor, IGF1R, and activates the PI3K/AKT pathway to positively regulate muscle protein synthesis and mTOR, whereas MSTN binds to its receptors, activin A receptor (ACVR) types I/II, to upregulate the SMAD3 pathway and negatively regulate mTOR and muscle protein synthesis (Schiaffino et al., 2013). The intramuscular injection of ANT22 upregulated mTOR but did not alter MSTN or IGF1.

The expression of myosin heavy-chain isoforms was not altered with AGO127 treatment. Muscle fiber histology found an increase in the cross-sectional area of type I and no change in type II fibers with AGO127 injection. MYHCI, MYHCIIA, and MYHCIIX were all upregulated with ANT22 injection. Changes in the color patterns of intermediate fibers were observed in the muscle fiber histology for ANT22. The injection of ANT22 increased the oxidative metabolism of the fibers toward more yellow–red type IIax fibers, whereas SHAM22 had greater size of more glycolytic fibers indicated by more green–yellow fibers. Others have shown that miR-22-3p inhibition enhances the fiber-type conversion from type II to type I fibers via the AMPK/SIRT1/PGC1α pathway in C2C12 muscle cells (Wen et al., 2021). Resveratrol is reported to alter miR-22-3p expression in C2C12 muscle cells and shift muscle fibers to more oxidative metabolism via the AMPK/SIRT1/PGC1α pathway (Wen et al., 2020; Wen et al., 2021). The benefits of transitioning to a more oxidative fiber with ANT22 may be related to the need for greater protein synthesis capacity, changes in signaling pathways, or altered protein degradation rates (van Wessel et al., 2010). In mammals, there is a muscle paradox for the size of highly oxidative fibers that can be attained before they become anoxic or convert to a more glycolytic metabolism (Lynch and Koopman, 2019). Others have shown that resveratrol supplementation in pigs also switches fiber type to a more oxidative state, which is regulated by the adiponectin signaling pathway and insulin sensitivity (Huang et al., 2020). Changes in insulin resistance in the skeletal muscle may alter lipid droplets, which could be related to the density of the skeletal muscle (Li et al., 2019). Additional research is needed to further elucidate how alterations in miR-22-3p expression alter organelles (mitochondria, lipid droplets, nuclei, etc.) in the skeletal muscle and the density of injected muscle regions observed in this study.

Proteomic analysis showed that NAGK was upregulated for AGO127-treated longissimus samples. NAGK is involved with amino sugar metabolism and is needed for cellular migration (Islam et al., 2021). TBRG4, a member of the FAST kinase domain-containing protein family, was downregulated in AGO127 samples. The FASTKD family is associated with mitochondrial respiration and is found abundantly in tissue rich in mitochondria, like skeletal muscle (Simarro et al., 2010). Proteomic analysis of ANT22-treated longissimus samples revealed that there was an upregulation of proteins associated with intracellular transports such as NRBP1 and TRAPPC2. NRBP1 is associated with endoplasmic reticulum and Golgi apparatus transport (De Langhe et al., 2002). TRAPPC2 is a tethering factor that assists in the movement of proteins between cellular compartments (Sacher et al., 2019). Additionally, there was a downregulation of ACSL3 protein which is involved in fatty acid uptake and can regulate lipogenesis by facilitating the activation of proliferator-activated receptor-γ (Yan et al., 2015). Long-chain acyl-CoA synthetases (ACSL) are involved in human skeletal muscle fat oxidation and storage (Stierwalt et al., 2021). ACSL3 is found in the endoplasmic reticulum and lipid droplet, whereas ACSL1 is present in the endoplasmic reticulum and mitochondria (Poppelreuther et al., 2023). Changes in ACSL abundance may limit the conversion of fatty acids to fatty acid acyl-CoAs, which may direct them to oxidation or storage as lipid droplets in skeletal muscle.

The use of mimic/agomiR and antagomiR technologies was successful in altering endogenous miRNA expression *in vitro* and *in vivo*. The upregulation of miR-127 or downregulation of miR22-3p enhanced myoblast proliferation *in vitro*. The use of agomiR-127 and antagomiR-22-3p *in vivo* with repeated intramuscular injections was effective in altering miRNA expression and upregulating KIF3B and ACVR2A mRNA targets, respectively. The injection of antagomiR-22-3p upregulated mTOR, a major regulator of protein synthesis, and altered muscle fiber metabolism to shift toward a more oxidative fiber. Additional research is needed to further examine dose levels, timing of injections, and other methods for delivering miRNA treatments to enhance muscle hypertrophy in IUGR lambs during early postnatal growth.

## Data Availability

The raw data supporting the conclusion of this article will be made available by the authors, without undue reservation.
