# Supplementary material for: Use of AgomiR and AntagomiR technologies to alter satellite cell proliferation in vitro, miRNA expression, and muscle fiber hypertrophy in intrauterine growth-restricted lambs
Source: Front Mol Biosci. 2023 Nov 3;10:1286890. doi: 10.3389/fmolb.2023.1286890 (PMC10656622; doi:10.3389/fmolb.2023.1286890)
Supplement: Supplementary file 1 [file Table2.DOCX]

**Supplemental Table 1.** Primer sequences for qPCR analyses of mRNA involved in satellite cell proliferation, target mRNA expression, protein synthesis and for normalization.

| **Gene** | **Forward, 5’-3’** | **Reverse, 5’-3’** | **Efficiency** |
| --- | --- | --- | --- |
| ACVR2a | GCGTTTGCCGTCTTTCTTATC | CCAGTGCGATTGGTTCTATCT | 0.94 |
| ACVR2b | GTCGTCTTGAGCTGTGTTATCT | CAGGTGTGTGCGGGTATTTA | 0.94 |
| AKT3 | GGACACTTCCTACGGACTAATG | CACTACCAGGAGCAAGATAAGG | 1.01 |
| AMPK | GTCAAAGTCGGCCAAATGAT | CCTCCGAACACGCAAATAAT | 0.99 |
| HDAC4 | GGGTCTCTTGTTTGTCTGTCTC | CACCGACTCCTGCTGTTTATT | 1.05 |
| IGF1 | TTGCACTTCAGAAGCAATGG | GGAGGATGTGACTGGCATCT | 0.95 |
| IGFBP3 | CAGAGCACAGACACCCAGAA | CACAGTTGGGAATGTGGATG | 1.02 |
| IGFBP5 | GGTTTGCCTGAACGAAAAGA | CTGGGTCAGCTTCTTTCTGC | 1.08 |
| KIF3B | GAGCAGGTGCTCGTTCTTATAG | GGAGGCAGAGGAAGTGTTATTT | 0.90 |
| MSTN | AGTACGATGTCCAGAGAGATGA | TATCCACAGTTGGGCCTTTAC | 1.02 |
| mTOR | GACCTTCTGCCTTCACAGATAC | CTCCTTCTTGACACAGCTTAGG | 1.05 |
| SEPT7 | GTATCCAGGTCCTTCCCATAGA | CCTACCACAGCAAGAGGTAAAC | 0.98 |
| S1PR3 | TTCTCGGATAGAGGAGCCTATT | CAGGGTTTCGTTGGAGGTATAA | 1.05 |
| SIRT1 | CAGGACTTTGGATTAGGGAGATG | CACGTTTCTACCCTCCTGAATAC | 0.97 |
| TGFBR1 | ACCAGGACCACTGCAATAAA | AGTGCGGTTATGGCAGATATAG | 1.05 |
| EIF3K | CTGACAGACAGCCAGCTAAA | CACGATGTTCTTGGGCTTAATG | 0.98 |
| UXT | CCACGGTTCTTACTAACACTTCT | TTGGCCAGCTGCTCATATAC | 0.91 |
