# Supplementary material for: Use of AgomiR and AntagomiR technologies to alter satellite cell proliferation in vitro, miRNA expression, and muscle fiber hypertrophy in intrauterine growth-restricted lambs
Source: Front Mol Biosci. 2023 Nov 3;10:1286890. doi: 10.3389/fmolb.2023.1286890 (PMC10656622; doi:10.3389/fmolb.2023.1286890)

Supplemental Figure 1. Image of isolated satellite cells stained for PAX7 (DSHB PAX7 antibody; red) and nuclei (Hoechst 33342; blue). Nine images were collected and analyzed with 98.5% staining for both PAX7 and Hoechst.

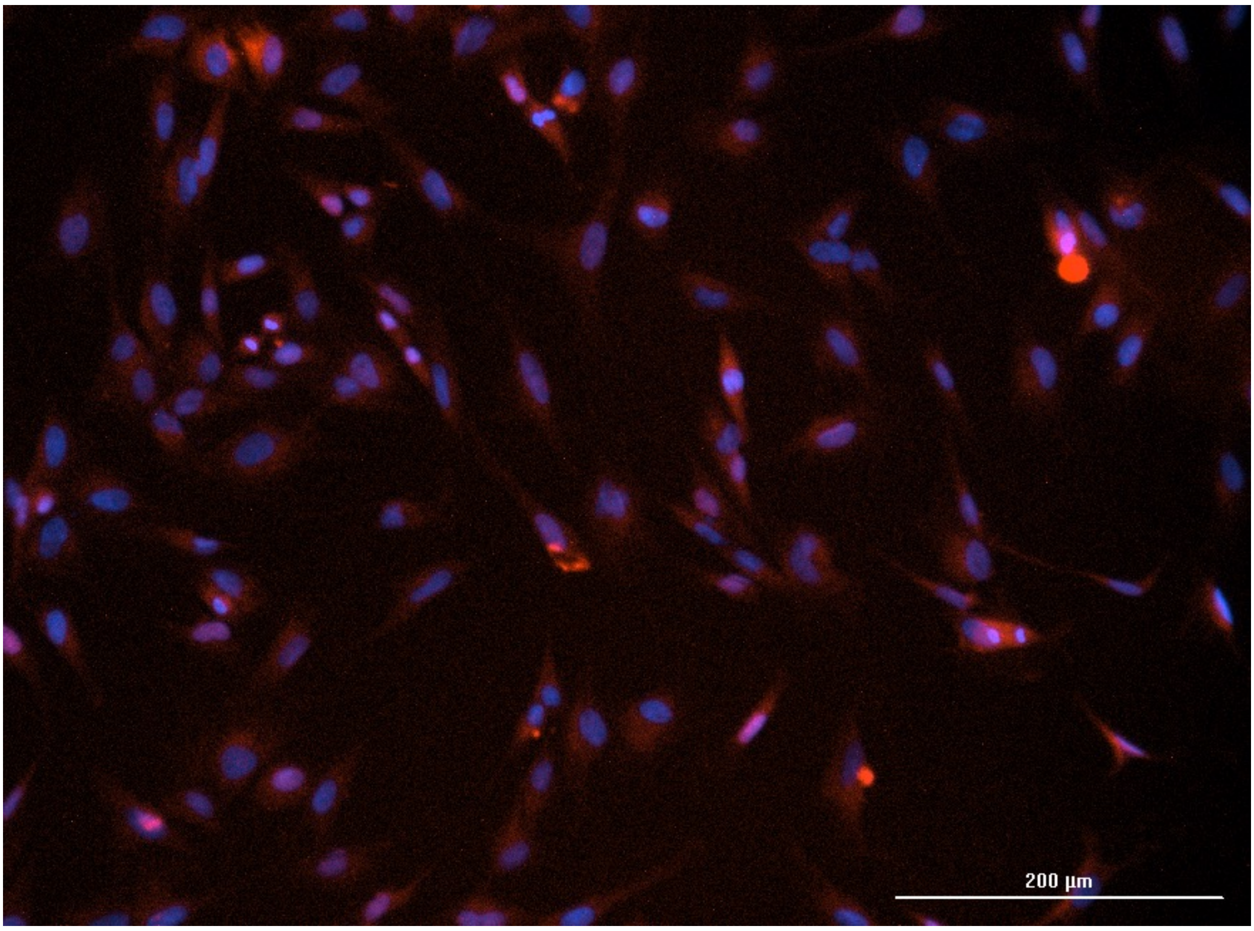

Supplement: Supplementary file 2 [file DataSheet1.PDF]
